# Impact of Bone Fracture on Ischemic Stroke Recovery

**DOI:** 10.3390/ijms19051533

**Published:** 2018-05-22

**Authors:** Meng Wei, Haiyian Lyu, Kang Huo, Hua Su

**Affiliations:** Center for Cerebrovascular Research, Department of Anesthesia and Perioperative Care, University of California, San Francisco, CA 94110, USA; wm.3686@stu.xjtu.edu.cn (M.W.); Haiyan.Lyu@ucsf.edu (H.L.); kang.huo@ucsf.edu (K.H.)

**Keywords:** bone fracture, stroke, cognitive decline, inflammation

## Abstract

Stroke is one of the most devastating complications of bone fracture, occurring in up to 4% of patients after surgical repair for hip fracture. Bone fracture and ischemic stroke have many common risk factors. The impact of bone fracture on stroke recovery has not drawn much attention in the research field. Bone fracture could occur in stroke patients at different times during the recovery phase, which steepens the trajectory of cognitive decline, greatly affects the quality of life, and causes a heavy burden on healthcare resources. In this paper, we reviewed the growing information on the pathophysiological mechanisms by which bone fracture may affect ischemic stroke recovery process.

## 1. Introduction

Up to 5% of stroke victims sustain a bone fracture [1,2,3,4,5,6]. The risk of stroke doubles after hip fracture [7,8], and remains high 10 years after hip fracture [9]. Clinical scenarios of this combination include stroke victims with bone-demineralization falling due to limb weakness resulting in bone fracture; alternatively, an initial bone fracture causes hemorrhagic shock and systemic inflammation that can precipitate an ischemic stroke. As advanced age is a risk factor for both stroke and fracture, it is imperative to understand the neurobiologic underpinnings for the association of these two conditions on the trajectory of aged-related cognitive decline. Stroke doubles the risk of developing dementia [10] and cognitive impairment occurs in nearly half of hip fracture patients [11]. The aging demographic change in the US will increase the burden of these diseases at both the individual and societal levels.

Bone fracture and stroke can occur in different sequences. Patients with both injuries will have less optimal outcomes than patients that have single injury. Although stroke and bone fracture have many common risk factors [5,12,13], strategies for preventing [14,15] or treating stroke may adversely influence bone healing [4].

Currently, there is no optimal strategy for prevention or treatment of post-stroke bone fracture or post-bone fracture stroke. Intravenous thrombolysis is widely accepted, and tissue plasminogen activator (tPA) is still the only drug approved by the U.S. Food and Drug Administration (FDA) for the management of acute ischemic stroke [16,17]. However, the therapeutic time window for tPA (<4.5 h post stroke) is quite narrow. Application of tPA beyond the therapeutic time window may produce hemorrhagic transformation (HT), which increases morbidity and mortality in stroke patients. Drugs that preserve the integrity of the blood-brain barrier (BBB) or enhance vascularization have been tested to reduce tPA-induced HT [18]. It has been shown that the basement membrane disintegrated and detached from the astrocyte endfeet in tPA-treated hypertensive rat model of middle cerebral artery occlusion (MCAO). Edaravone, approved by the FDA recently to treat patients with amyotrophic lateral sclerosis, prevented the dissociation of the neurovascular unit, dramatically decreased the HT, and improved the neurologic score and survival rate of the tPA-treated rats [19]. G-CSF can also attenuate delayed tPA-induced HT [20]. With the improvement of the technologies, the therapeutic window for ischemic stroke is extending. For example, the window for thrombectomy has been extended from 6 h to 24 h lately [21]. Other therapeutic strategies, such as stem cell therapy, can extend the stroke therapy window further [22].

For most individuals who have not received thrombolysis, antiplatelet or anticoagulation, therapy is recommended to decrease the incidence of recurrent stroke [16,23]. However, thrombolysis or anticoagulation therapies can increase the incidence of bone fracture hemorrhage, and there are no guidelines currently on the use of antithrombotic drugs for bone fracture patients [24].

Stroke and bone fracture share some common risk factors, such as hypertension and diabetes mellitus [5,12]. Management of these risk factors to prevent stroke or bone fracture is complicated. For example, using pioglitazone (a drug in the thiazolidinedione class of peroxisome proliferator-activated receptor γ [PPAR-γ]—one of the most potent insulin-sensitizing drugs) to treat insulin-resistant non-diabetes patients who have a history of stroke reduced the risk of stroke, but increased the risk of bone fracture [14,15].

Despite the growing number of patients with both injuries, few studies have been designed to address the impact of bone fracture/surgery on stroke recovery, which, if unabated, will lead to an increased burden of these illnesses on individual suffering and healthcare resources. Due to the lack of biological understanding of the interaction of these two conditions, there are no specific interventions to prevent post-bone fracture/surgery stroke, nor post-stroke bone fracture. Understanding the underlying mechanism could lead to the discovery of new targets for the development of innovative therapies to improve the recovery of patients with stroke and bone fracture and reduce the health care costs.

## 2. Bone Fracture Causes Hippocampal Inflammation and Cognitive Dysfunction

Peripheral trauma strike and surgery commonly cause mild transient cognitive impairment among young and otherwise healthy patients [25,26]. However, long-term cognitive decline can occur in patients with some risk factors, such as advanced age [27,28]. Advance age is the major risk factor for the development of cognitive impairment and dementia [26,29,30]. Compared to other hospitalized groups, cognitive dysfunction is present more often in orthopedic patients [31,32]. The elderly are prime candidates for hip- and/or knee-replacement surgeries, partly due to increased rates of age-related bone fragility [33]. It has been estimated that 25–40% of the patients undergoing non-cardiac surgery develop cognitive decline by the time of hospital discharge. The risk of dementia remains significantly higher (>10%) among patients over 60 years old at 3 months after surgery [34,35].

Patients with Metabolic Syndrome appeared to have a greater likelihood of having impaired cognitive function after either cardiac or non-cardiac surgery [36]. For patients with preexisting cognitive decline—such as Alzheimer’s disease, or who are at risk for developing it—peripheral trauma and surgery can deteriorate cognitive function and exacerbate neurodegeneration [37,38,39].

Gender factors also predispose to this post-trauma cognitive decline [40]. One longitudinal cohort study on hip fracture patients found that poor functional outcomes were related to the baseline pre-fracture dementia and cognitive impairment during hospitalization, which was more like to occur in aged males [41].

Animal studies show that aseptic long bone fracture can cause neuroinflammation and cognitive decline. In healthy, young animals, inflammatory cytokines, such as tumor necrosis factor-alpha (TNFα), interleukin-1β (IL-1β) and Interleukin-6 (IL-6), are increased in peripheral blood and hippocampus after aseptic tibia fracture, which is associated with short-term memory dysfunction [42].

Peripheral blockade of TNFα with antibody attenuated the increase of hippocampal IL-1β after tibia fracture, suggesting that it is an upstream mediator [42]. Furthermore, the activation of TNFα/nuclear factor (NF)-κB signaling pathway impairs the integrity of BBB, which increases peripheral macrophage infiltration into the hippocampus. All of these interfere with the processes required for memory and learning [43,44].

Animals with risk factors suffer a longer period of cognitive dysfunction after peripheral surgery [45]. The memory deficit in rats that were selectively bred for low exercise endurance to mimic metabolic syndrome lasted more than 5 months post-operatively [38]. Our recent study found that the spatial memory dysfunction in adult mice subjected to tibia fracture shortly before stroke lasted beyond 8 weeks.

## 3. Bone Fracture Exacerbates Ischemic Stroke Injury

Animal studies have shown that bone fracture shortly before or after ischemic stroke exacerbates stroke injuries. Mice that had tibia fracture 6 h before, one day before, or one day after the permanent occlusion of distal middle cerebral artery (pMCAO) had more severe behavior deficits than mice subjected to pMCAO alone. They took a longer time to remove the adhesive on the fore paw opposite to the stroke side in the adhesive removal test, and made more turns to the stroke side during the corner test [46,47,48]. The mice with bone fracture and pMCAO have larger infarct volumes and more apoptotic neurons in the peri-infarct region than mice with stroke alone [46,47,48].

Tibia fracture occurring shortly before or after ischemic stroke enhanced inflammation in the peri-infarct areas [46,47,48]. Mice with both injuries had higher levels of IL-6 in the ipsilateral hemisphere [46,48], and more CD68^+^ cells in the peri-infarct regions compared to mice with pMCAO only [46,48]. Both bone marrow-derived macrophages and active microglia are increased in mice subjected to both injuries [46,48]. Intraperitoneal administration of a high-mobility group box 1 protein (HMGB1)-neutralizing antibody attenuated fracture-enhanced infarct volume, apoptotic neurons in the infarct area, the number of CD68^+^ cells in the peri-infarct region and behavioral dysfunction [46]. Using clodrolip to selectively deplete macrophages significantly reduced the number of CD68^+^ cells in the peri-infarct region, the infarct volume and neurobehavioral dysfunction [46]. Microglia and macrophages can polarize into type I (pro-inflammation, M1) and type II (anti-inflammation, M2) phenotypes [49,50,51]. Tibia fracture increased the number of macrophages/microglia expressing M1 markers (iNOS and CD11b) and reduced the number of macrophages/microglia expressing M2 markers (CD206, and IL-10) [47,52]. These experiments indicated that tibia fracture enhanced neuroinflammation during the acute stage of stroke.

Oxidative stress participated in the pathogenesis of ischemic or hemorrhagic stroke brain injuries [53,54]. This process involves nicotinamide adenine dinucleotide phosphate (NADPH) oxidase [55], which is regulated by the inflammatory transcription factor, NF-κB [56]. Tibia fracture reduced the expression of anti-oxidant genes [superoxide dismutase 1 (SOD1) and glutathione peroxidase 1 (GPX1)] and increased phospho-NF-κB p65 positive microglia/macrophages and the expression of the two subunits of pro-oxidative stress protein NADPH oxidase (gp91^phox^ and p22^phox^) in pMCAO and pMCAO plus tibia fracture mice [47,52].

Monoamine oxidase B (MAOB) is a type of MAO that is linked to oxidative stress in tissues [57]. Mice with tibia fracture one day after the pMCAO had more MAOB-positive astrocytes compared with the pMCAO-only mice, which was associated with an increase of brain edema [58].

## 4. Inhibition of Inflammation Could Reduce the Negative Impact of Bone Fracture on Ischemic Stroke Recovery

Patients suffering stroke and bone fracture often have unfavorable outcomes. In order to improve the quality of life and cut down on the medical costs for patients suffering both injuries, novel therapies are urgently needed. Animal studies show that neuroinflammation is one of the underlying mechanisms of the negative impact of bone fracture on stroke recovery. In this section, we will review some anti-inflammation strategies that have been tested in animals with bone fracture and stroke.

HMGB1 is a non-histone DNA-binding protein that is widely expressed in various tissues, including brain [44,59]. HMGB1 is among the earliest-activated cytokines after surgical trauma, causing neuroinflammation and cognitive impairment [44]. In adult mice, plasma levels of HMGB1 increased within 1 h after tibia fracture [44]. Many inflammatory conditions, such as aseptic trauma, acute lung injury, arthritis and stroke engage the innate immune response through the passive release of HMGB1 after cell lysis at sites of tissue damage [60]. By interaction with pattern-recognition receptors (PRRs) on circulating bone marrow-derived monocytes, HMGB1 induces the synthesis and release of pro-inflammatory cytokines including TNFα through the transcriptional upregulation of NF-κB [61], disrupting BBB integrity and allowing the monocyte to infiltrate into the brain [44]. Concurrently, studies show that HMGB1 increases expression of the monocyte chemoattractant protein-1 (MCP-1) in the central nervous system [62]. Therefore, HMGB1 plays a key role in initiating the inflammatory cascade after bone fracture.

Animal studies showed that neutralizing HMGB1 using an HMGB1-specific antibody significantly ameliorated brain injury and neuronal deficits in rats subjected to MCAO [63]. Anti-HMGB1-neutralizing antibody decreased the BBB permeability of tibia fracture mice through time-dependent inhibiting morphological changes of astrocyte and endothelial cells [60]. Neutralizing HMGB1 also prevented the negative impact of tibia fracture on ischemic stroke injury, reducing infarct size, behavioral dysfunction and macrophage/microglia infiltration [46].

IL-1β is also a key mediator of inflammatory response to trauma [64]. IL-1β contributes to both innate and adaptive immunity [65]. Increasing plasma concentration of IL-1β after peripheral trauma, such as tibia fracture, exerts concentration-dependent effects on hippocampal-dependent memory dysfunction, and accentuates adverse neurological behavior outcomes and brain injury after ischemic stroke [44]. The increase of BBB permeability during this process may due to the suppression effects of IL-1β on sonic hedgehog (SHH) expression in astrocytes and downregulation of tight junction (TJ) protein expression in endothelial cells [66,67]. Therefore, IL-1β could represent an attractive therapeutic target to attenuate brain injury and cognitive decline caused by bone fracture.

It has been shown that pre-treatment with IL-1 receptor antagonist or genetically knockout of IL-1 receptor mitigates the level of circulating IL-1β and hippocampal microgliosis in tibia fracture mice [42]. Systemic infusion of anti-IL-1β monoclonal antibody after cerebral ischemia insult attenuates the increase of IL-1β after ischemic incidence and BBB permeability [68]. Increased central IL-1β activity impairs the consolidation of memories that depend on the hippocampal function but have no effect on the consolidation of hippocampal-independent memories [69]. Conversely, IL-1 has also been suggested to facilitate hippocampal-dependent learning and memory; the IL-1 type one receptor (IL-1RI) null mice have deficits in tasks of visuospatial learning and memory [70]. These results may argue for a more careful and in-depth evaluation of the roles of endogenous IL-1 in hippocampal-independent cognitive decline.

The cholinergic pathway has been found to inhibit cytokine release through a mechanism that requires the α7 subunit-containing nicotinic acetylcholine receptors (nAChRs) [71]. Stimulating the cholinergic anti-inflammatory pathway by electrical or pharmacological methods significantly suppresses the systemic levels of TNF and other pro-inflammatory cytokines during endotoxemia [72,73,74]. The α7 nAChR subunit expressed on the surface of macrophages regulates this pathway during inflammation [75]. Activation of α7 nAChR in macrophages dampens the inflammatory response.

It has been shown that *N*-Methyl_d-aspartic acid (NMDA) receptor activation-induced excitoxicity was avoided in rat neocortical neurons when dimethoxybenzylidene anabaseine (DMXB), the α7 nicotinic acetylcholine receptor agonist, was added 24 h before NMDA addition. Unlike muscarinic antagonists, nicotinic antagonists were able to nullify this neuroprotection. Additionally, when rats were subjected to focal ischemic insults, infarct sizes were smaller in rats that have received DMXB 24 h beforehand. Interestingly, these neuroprotective effects were not present when DMXB was added simultaneously with either NMDA or focal ischemic insults. In a non-apoptotic model, α7 receptors seemed to provide neuroprotection when mecamylamine was absent. Ultimately, the results imply that α7 receptors contribute to neuroprotection for ischemic stroke, and that a mechanism exists in which intracellular calcium ion concentrations temporarily increase via α7-activation and are accompanied by protein kinase C (PKC) activation.

Activation of the nAChR protects against ischemic stroke-related cerebral damage [76,77,78] and reduces tibia fracture-induced systemic/hippocampal inflammation. It has been shown that ischemic stroke is associated with the activation of the vagal cholinergic anti-inflammatory pathway [79,80], which protects against ischemic stroke-related cerebral damage [76,77]. In addition, α7 nAchRs protect against glutamate neurotoxicity and neuronal ischemic damage [78].

Activation of α7 nAChR reduces brain damage in a mouse intracerebral hemorrhage model [81] and in a rat subarachnoid hemorrhage model [82]. Treating the mice with α7 nAChR agonists attenuates the cognitive decline and hippocampal inflammation caused by bone fracture [83]. Treating mice with a selective α7 nAChR agonists, PHA (PHA568487), reduced neuronal injury and functional deficits of mice subjected pMCAO [52] or bone fracture one day after pMCAO [47]. PHA-treated mice had fewer apoptotic neurons and CD68^+^ cells in the peri-infarct region. PHA treatment also decreased oxidative stress and the number of pro-inflammatory macrophages (M1), and increased the number of pro-recovery macrophage (M2). In addition to inhibiting inflammation, PHA treatment improved BBB integrity and reduced MAOB-positive astrocytes and brain edema in mice subjected to bone fracture one day after pMCAO [58]. Thus, modulating α7 nAchR activity could be developed into a novel treatment to reduce the adverse influence of bone fracture on recovery in stroke patients.

However, it is well known that inflammation has bi-physiological roles in stroke recovery [84,85]. Simply inhibiting inflammation may not improve long-term outcomes of stroke patients [86]. Therefore, the timing, dose and the types of anti-inflammatory agents have to be tested carefully before applying to patients.

## 5. Milestone Studies in Understanding Bone Fracture and Stroke Interplay

Important investigations carried out by various laboratories over the past decade have contributed to our understanding of how bone fracture may impact stroke pathology (Table 1).

Attempting to connect peripheral trauma to neurological decline, a group studied orthopedic surgery mouse models to probe the mechanisms underlying postoperative cognitive dysfunction (POCD) [42]. Specifically, they examined whether memory deficits and hippocampal inflammation were initiated by surgical trauma-generated systemic inflammation. The researchers performed tibia surgery on both interleukin-1 receptor knockout mice (IL-1R^−/−^) and wild-type mice, analyzed the animals’ memory capabilities via fear-conditioning tests, and measured systemic and hippocampal cytokines and activated microglia. They found that the mice were afflicted with memory problems, elevated plasma cytokines, and increased hippocampal reactive microgliosis and IL-1β transcription and expression. Surgery-related memory deficits and neuroinflammation were alleviated with minocycline treatment and inhibition of IL-1β in both IL-1R^−/−^ mice and mice given an IL-1R antagonist. Thus, they concluded that cognitive dysfunction following surgery is produced by IL-1β-controlled hippocampal inflammation, which is elicited by an innate immune response to peripheral surgical intervention.

The follow-up experiment demonstrated that HMGB1 increased immediately after bone fracture and is an important mediator for post-bone fracture cognitive dysfunction [43]. Further probing the potential overlap of systemic and central inflammatory pathways, which could contribute to bone fracture-induced cognitive pathology, the authors employed a monoclonal antibody (mAb) against HMGB1. They explored how anti-HMGB1 would exert neuroprotection when HMGB1 translocates within the brain, when the blood-brain barrier (BBB) is disrupted, and when the brain experiences edema. Moreover, the justification for this experiment was based on a past study by the same authors in which rats with MCAO had abated brain infarcts when the same mAb was injected intravenously. Rats were subjected to MCAO surgery to produce ischemic insult and via intravenous injection, were either given anti-HMGB1 or a control IgG antibody. In MCAO rats, HMGB1 was released by neurons and translocated within the brain in a time-sensitive manner. The anti-HMGB1 lessened brain edema and inhibited the opening of inter-endothelial cell tight junctions, astrocyte end feet swelling and detachment from the basal lamina. Additionally, BBB permeability was enhanced and endothelial cells and pericytes underwent morphological changes when HMGB1 was incorporated in an in vitro BBB model. The HMGB1 mAb successfully restricted these effects and led to dwindling HMGB1 serum levels. Hence, ischemic stroke deficits could potentially be attenuated with the anti-HMGB1 mAb, as demonstrated by the antibody’s ability to remove HMGB1, stymie brain edema, and safeguard the BBB.

A study that induced tibia bone fracture one day after pMCAO in mice found an increase in infarct volume, more severe neurobehavioral abnormalities, enhanced immune response, and increased neuronal death when compared to stroke-only animals. This study probed the implications of HMGB1 [46], reporting that intraperitoneal injection of HMGB1 in stroke-only animals mimicked the bone fracture effects and increased the macrophage infiltration of stroke-only animals, whereas HMGB1 neutralization nullified the fracture-induced injury exacerbation in dual-injury animals. Authors concluded that post-stroke bone fracture enhances macrophage infiltration via an HMGB1-dependent mechanism, and that HMGB1 may be a promising target in minimizing the adverse consequences of bone fracture following stroke.

In addition, activation of the α7 nAChR using it specific agonist PHA568487 (1) reduced CD68^+^ microglia/macrophages, (2) shifted microglial/macrophage polarization from M1 to M2, (3) reduced apoptotic neurons, and (4) lessened oxidative damage in mice subjected to tibia fracture followed by pMCAO one day later [47]. More specifically, authors found that that PHA568487 inhibits NF-κB phosphorylation, down-regulates its activity, and lessens oxidative gene expression—all consistent with their previous finding that PHA inhibits TNFα-induced NF-κB activation. Authors thus conclude that α7 nAChR plays an important protective role in the stroke brain with and without tibia fracture.

In probing the effects of circulating pro-inflammatory cytokines on BBB permeability, a study found that IL-1β contributes significantly to the loss of BBB integrity following stroke. By designing an IL-1β-neutralizing antibody, this group demonstrated that intravenous administration of the anti-IL-1β antibody was able to preserve BBB integrity following experimental stroke, although the exact mechanisms of this change were not identified. Specifically, authors did not observe widespread alterations in the expression of tight junction proteins such as Occludin, Claudin-1/5, or ZO-1/2 across various brain regions. Nonetheless, identifying systemic causative agents for increased BBB permeability has important implications in our understanding of how systemic injuries may worsen concurrent stroke pathology.

Finally, a recent study connected multiple of the concepts discussed thus far by identifying the ability of α7 nAChR activation with PHA568487 to reduce bone fracture-induced exacerbation of BBB permeability following pMCAO stroke. Tibia fracture induced 1 day after stroke resulted in increased brain water content and MAO-B-positive astrocytes, while PHA568487 treatment reversed these effects and increased Claudin-5 expression. Furthermore, α7 nAChR inhibition with methyllycaconitine resulted in the opposite effects. Taken together, this study indicates that in addition to the previously mentioned mechanism of α7 nAChR, preservation of BBB integrity and decreased oxidative/astrocytic damage may contribute to its protective properties [58].

## 6. Clinical Implications

Stroke is not only the fourth-leading cause of death in the United States [87], but is also an important risk factor for bone fracture [6]. Stroke is also one of the most devastating complications of bone fracture, occurring in 4% of patients in the first year after hip surgery [8]. Bone fracture patients with post-fracture stroke have poor functional recovery and require more care in the first year after bone fracture than those who do not have stroke [31]. The overall number of hip fractures is high and is anticipated to exceed 6 million a year [32], which could increase with the increases of aged population. The lifetime risk of hip fracture is 17.5% for women and 6% for men [33,88]. Hip fracture patients are at a 1.54-fold higher risk of developing stroke than controls [89], at an incidence of 64.6 per 1000 persons per year [89]. The estimated occurrence of stroke after hip fracture is 64,600 per year.

Stroke and bone fracture share some common risk factors, such as hypertension and diabetes mellitus [5,12]. However, management of these risk factors to prevent stroke or bone fracture is contradictory. Stroke doubles the risk of developing dementia [10]. Cognitive impairment occurs in nearly half of hip fracture patients [11]. Patients with both injuries will have worse outcomes than patients that have single injury. How bone fracture increases ischemic injury in the brain is largely unknown. Understanding the underlying mechanisms could lead to the discovery of new targets for developing innovative therapies to improve the recovery of patients with stroke and bone fracture and reduce the health care costs.

## 7. Future Directions

We have discussed the potential impact of bone fracture on stroke recovery and the underlying mechanisms. However, there are still some questions that need to be clarified; for example, the incidence of post-bone fracture cognitive dysfunction in humans needs more investigation.

Surgery remains the most effective treatment method for bone fracture, and due to the cognitive dysfunction associated with glucocorticoid exposure, anesthesia type [90,91] and surgery procedures [92], it is important to avoid potential secondary injury among stroke patients. Although increasing evidence shows that inflammatory and immune activities play important roles in the decline of cognitive function of bone fracture patients, and some human trials have reported that anti-inflammation decreases the frequency of post-operative cognitive dysfunction, the selection of correct anti-inflammation agents needs to be made cautiously, because some agents can cause unwanted side effects [93].

In addition, bone fracture causes acute pain and chronic pain, which can last long after the fracture. A multivariate regression analysis revealed that post-operative analgesia was associated with the development of post-operative cognitive dysfunction [94]. Therefore, the impact of pain on stroke recovery needs to be studied in the future.

In summary, numerous studies over the past decade have reported the impact of bone fracture on cognitive function and stroke recovery. However, the mechanism remains largely unknown. We anticipate that more vigorous and longitudinal studies would help to uncover the underlying mechanisms and develop new intervention strategies to improve the recovery of patients with stroke and bone fracture.

## Figures and Tables

**Table 1:** Milestone Discoveries.

Year	Authors	Major Discovery
2010	Cibelli et al.	TNF-α an upstream mediator for hippocampal IL-1β following tibia fracture
2010	Terrando et al.	HMGB1 initiate inflammatory cascade post-bone fracture which causes cognitive dysfunction
2013	Degos et al.	HMGB1 antibody reduces cell death in a fracture-enhanced stroke
2014	Han et al.	α7 nAChR agonists diminishes microglia/macrophage infiltration of the peri-infarct region
2014	Vacas et al.	HMGB1 antibody decreased BBB permeability after fracture-enhanced stroke
2016	Zou et al.	PHA treatment attenuated brain edema and enhanced BBB integrity in fracture-enhanced stroke
